# Phylotype resolved spatial variation and association patterns of planktonic *Thaumarchaeota* in eastern Chinese marginal seas

**DOI:** 10.1007/s42995-023-00169-y

**Published:** 2023-04-07

**Authors:** Jiwen Liu, Fuyan Huang, Jiao Liu, Xiaoyue Liu, Ruiyun Lin, Xiaosong Zhong, Brian Austin, Xiao-Hua Zhang

**Affiliations:** 1 Frontiers Science Center for Deep Ocean Multispheres and Earth System, and College of Marine Life Sciences, Ocean University of China, Qingdao, 266100 China; 2 Laboratory for Marine Ecology and Environmental Science, Laoshan Laboratory, Qingdao, 266237 China; 3 Institute of Evolution and Marine Biodiversity, Ocean University of China, Qingdao, 266003 China; 4 Key Laboratory of Marine Chemistry Theory and Technology, Ministry of Education, Qingdao, 266100 China; 5 Institute of Aquaculture, University of Stirling, Stirling, FK9 4LA Scotland UK

**Keywords:** *Thaumarchaeota*, Phylotype, Association pattern, Spatial variation, Surface microlayer, Chinese marginal seas

## Abstract

The majority of marine ammonia oxidizers belong to *Thaumarchaeota*, a phylum of Archaea, which is distributed throughout the water column. Marine surface waters contain distinct thaumarchaeotal phylotypes compared to the deeper ocean, but spatial dynamics of the surface-associated lineages are largely unsolved. This study of 120 seawater samples from the eastern Chinese marginal seas identified contrasting distribution and association patterns among thaumarchaeotal phylotypes across different dimensions. Horizontally, *Nitrosopumilus*-like and *Nitrosopelagicus*-like phylotypes dominated the surface water (3 m) of the Yellow Sea (YS) and East China Sea (ECS), respectively, along with increased abundance of total free-living *Thaumarchaeota* in ECS. Similar compositional changes were observed in the surface microlayer. The spatial heterogeneity of particle-attached *Thaumarchaeota* was less clear in surface microlayers than in surface waters. Vertically, the *Nitrosopelagicus*-like phylotype increased in abundance from surface to 90 m in ECS, which led to an increase in the proportion of *Thaumarchaeota* relative to total prokaryotes. This occurred mainly in the free-living fraction. These results indicate a clear size-fractionated niche partitioning, which is more pronounced at lower depths than in the surface water/surface microlayer. In addition, associations of *Thaumarchaeota* with other microbial taxa varied between phylotypes and size fractions. Our results show that a phylotype-resolved and size-fractionated spatial heterogeneity of the thaumarchaeotal community is present in surface oceanic waters and a vertical variation of the *Nitrosopelagicus*-like phylotype is present in shallow shelf waters.

**Supplementary Information:**

The online version contains supplementary material available at 10.1007/s42995-023-00169-y.

## Introduction

*Thaumarchaeota*, which is a phylum of Archaea established in 2008 (Brochier-Armanet et al. 2008), plays important roles in nitrogen and carbon cycling by means of ammonia oxidation, carbon fixation (Könneke et al. 2005), and nitrous oxide production (Santoro et al. 2011). Members of *Thaumarchaeota* comprise all currently known ammonia-oxidizing archaea, and are recognized to be dominant in the coastal and open oceans, occupying both pelagic and benthic environments (Francis et al. 2005). In marine waters, the abundance of *Thaumarchaeota* varies greatly across water depths, with significantly lower cell numbers in the surface layer compared with the deep ocean. In the deep ocean, *Thaumarchaeota* accounts for up to ~ 40% of the total microbial population (Karner et al. 2001).

The depth-related shift in *Thaumarchaeota* abundance is accompanied by clear divergence in phylotype. The *amoA* gene-based phylogeny reveals two main lineages/phylotypes of ammonia-oxidizing archaea (AOA), i.e., water column A (WCA, *Nitrosopelagicus*-like AOA) and water column B (WCB) that prevail in the surface and deep seawater, respectively (Francis et al. 2005; Sintes et al. 2013; Smith et al. 2014a). Also, congruent phylotype differentiation has been revealed by 16S rRNA gene analysis (Reji et al. 2019; Tolar et al. 2020). Such a depth-dependent spatial separation reflects the varying adaptations of *Thaumarchaeota* to environmental changes, including light (Horak et al. 2018; Merbt et al. 2012), ammonium ions (Sintes et al. 2013), and oxygen (Molina et al. 2010; Qin et al. 2017). For example, the surface phylotype WCA contains photolyase to mitigate UV light exposure (Luo et al. 2014), whereas the deep phylotype WCB has a higher affinity for ammonia (Sintes et al. 2013). Furthermore, phylotype-specific association patterns have been observed (Reji et al. 2019). This suggests an important role of functional coupling and/or cross-feeding in governing *Thaumarchaeota* distribution patterns. The *Nitrosopumilus*-like lineage is the other phylotype that dominates the surface ocean and encompasses most of the cultivated marine *Thaumarchaeota* (Alves et al. 2018). It has also been shown to contain photolyases that may confer resistance to UV radiation (Bayer et al. 2016). Despite the widely recognized dominance of WCB below the euphotic zone, the balance between *Nitrosopelagicus*-like and *Nitrosopumilus*-like phylotypes in the surface ocean remains largely unresolved (Tolar et al. 2020).

Genomic evidence has revealed that *Nitrosopelagicus* may be more adapted to oligotrophic conditions than *Nitrosopumilus* (Santoro et al. 2015). This indicates that they may adopt different ecological strategies, including different efficiency of resource utilization. Only a comparatively few studies have reported on archaeal niche separation between size fractions, which consistently show that free-living *Thaumarchaeota* is more abundant (Smith et al. 2013; Zhong et al. 2020) and has different community compositions (Jing et al. 2018; Ijichi et al. 2019) compared to their particle-attached counterpart. This means that most members of *Thaumarchaeota* prefer a free-living lifestyle. However, the discovery of a thaumarchaeotal strain with the capacity to move toward nutrient-rich particles suggests the existence of a particle-attached strategy (Bayer et al. 2016). Maybe, there is divergence in the preferred lifestyle between *Thaumarchaeota* phylotypes and perhaps free-living and particle-attached *Thaumarchaeota* show different dynamic patterns, which are poorly characterized. This information is important for a comprehensive understanding of the dynamics of *Thaumarchaeota* in the marine environment.

The sea surface microlayer (SML), which is a boundary layer at the air–sea interface, is generally considered to comprise the uppermost 1 mm of the ocean (Cunliffe and Murrell 2009). The role of SML in regulating air–sea gas exchange has stimulated broad attention in investigating the ecosystem therein (Carlucci et al. 1985; Cunliffe et al. 2011, 2013; Maki. 2002). Physicochemical and microbiological characterization of SML has demonstrated enriched particles, organic matter, inorganic nutrients, and cell numbers in comparison with the underlying surface water (Cunliffe et al. 2011; Franklin et al. 2005). Also, contrasting microbial communities between SML and surface water have been observed, with the differences being more conspicuous in bacterial than archaeal communities (Cunliffe et al. 2009). Considering the higher concentration of inorganic nutrients (e.g., ammonia), we hypothesized that the SML may have selected differential thaumarchaeotal communities and nitrification activity compared to that of the surface water. However, the diversity and distribution pattern of *Thaumarchaeota* in marine SML remains largely unknown (Wong et al. 2018).

**Fig. 1:**
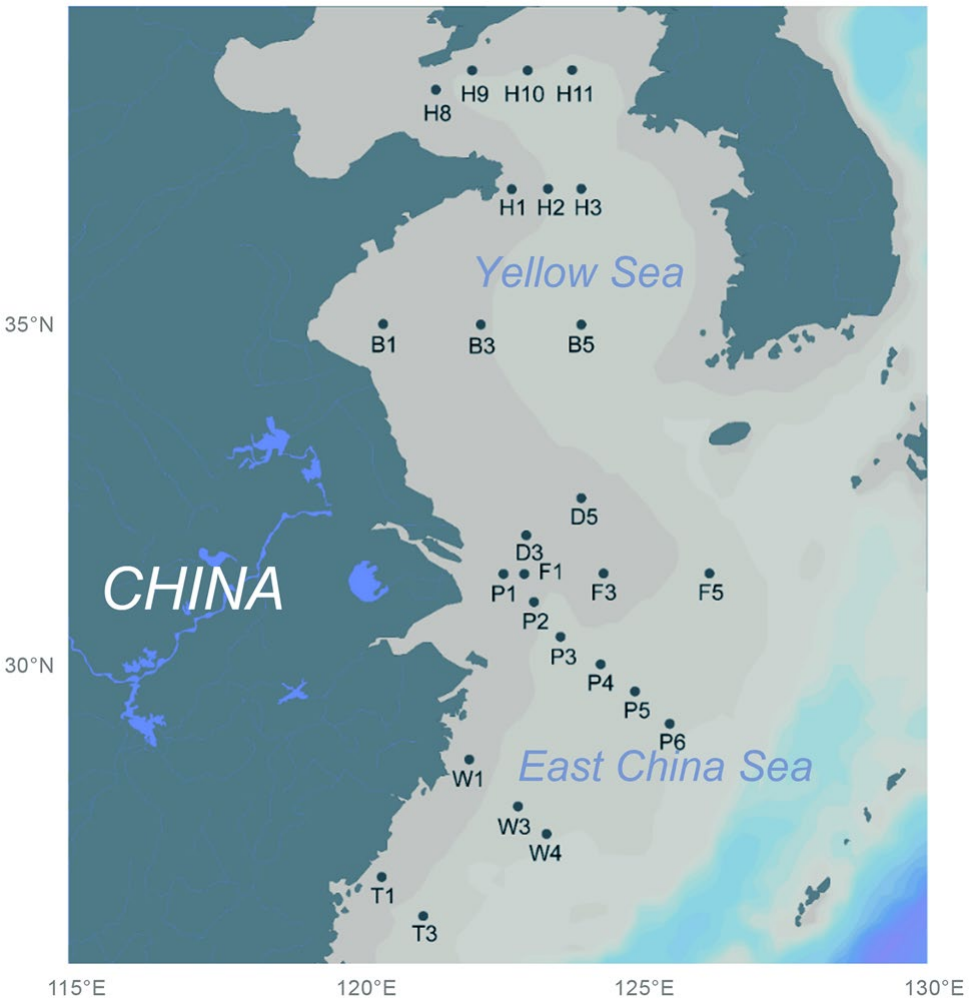
Map, which was generated in Ocean Data View, showing the sampling sites

Marginal seas host abundant *Thaumarchaeota* that experiences significant fluctuations in community composition in response to strong physicochemical gradients (Hu et al. 2011; Liu et al. 2014, 2018; Smith et al. 2014b). These coastal pelagic environments, thus, provide an interesting opportunity for studying the spatial dynamics of *Thaumarchaeota* and their niche partitioning between different size fractions. In this study, a multidimensional distribution pattern of the thaumarchaeotal community was investigated in the eastern Chinese marginal seas, where terrestrial discharge, anthropogenic activity, and ocean current cause significant environmental variability. The spatial distribution of *Thaumarchaeota* was examined across different sea areas (Yellow Sea [YS] *versus* East China Sea [ECS]), depths (surface [3 m] *versus* SML and lower euphotic depths [10–90 m]), and size fractions (free living *versus* particle attached). A total of 120 water samples were collected at 26 sites (Fig. 1 and Supplementary Table S1) and were subject to 16S rRNA gene sequencing and qPCR analysis. The results support the importance of analyzing the thaumarchaeotal community at a finer phylotype level, and provide a better understanding of the spatial heterogeneity of *Thaumarchaeota* in the surface ocean.

## Results

### *Thaumarchaeota* abundance and phylogeny

**Fig. 2:**
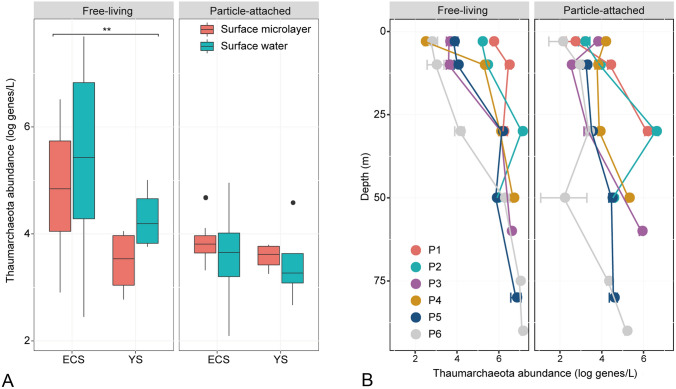
qPCR-derived *Thaumarchaeota* abundance in water samples of the eastern Chinese marginal seas. **A** free-living and particle-attached *Thaumarchaeota* in surface microlayer and surface water. **B** free-living and particle-attached *Thaumarchaeota* at different water depths of ECS sites P1–P6. *ECS* East China Sea; *YS* Yellow Sea. Samples from sites H1, H10, B1, B3, B5, and T3 were not size fractionated, and were not included. The asterisks denote significant differences between areas. **, *P* < 0.01

Quantitative analysis revealed that the abundance of thaumarchaeotal 16S rRNA genes varied from 1.24 × 10^2^ (except for the P5 particle-attached surface sample where no abundance was detected) to 4.9 × 10^7^ genes/L seawater across all samples (sample H2 was excluded due to abnormal high values of about orders of magnitude higher than other samples). Spatially, clear variations were observed between sampling areas, with higher gene abundance in the ECS than in the YS (Welch *t*-test, *P* = 0.004). The higher thaumarchaeotal abundance in the ECS mainly comprised the free-living fraction (Welch *t*-test, *P* = 0.003; Fig. 2A). Specifically, the free-living *Thaumarchaeota* had a high average abundance, approximately 20-fold higher than the particle-attached ones across the surface and SML samples. No significant differences were observed between the surface and SML samples (Welch *t*-test, *P* = 0.412; Fig. 2A). However, quantitative analysis revealed an increasing trend in *Thaumarchaeota* abundance with depth at sites of the ECS P section, with significantly higher abundance in the deepest layers compared to the surface zone across all sites (Welch *t*-test, *P* < 0.001; Fig. 2B). This depth-related variation seemed to be more pronounced in free-living *Thaumarchaeota* compared to its particle-attached counterpart, although the differences were not significant (Welch *t*-test, *P* = 0.743). However, in line with that observed in the surface water and SML, a higher abundance of free-living relative to particle-attached *Thaumarchaeota* was seen across the lower depths (Welch *t*-test, *P* < 0.001).

Illumina sequencing generated 5,962,278 high-quality reads of the 16S rRNA gene amplicons (average length of ~ 273 bp), which were rarefied to 28,134 per sample for equalizing sampling depth. A total of 6318 prokaryotic OTUs were clustered at a 97% similarity cutoff. Among them, 123 were affiliated to Archaea, corresponding to 1.95% of all OTUs and 4.15% of all sequences. The archaeal community comprised five phyla including *Thaumarchaeota* (33 OTUs), *Euryarchaeota* (55 OTUs), *Bathyarchaeota* (9 OTUs), *Woesearchaeota* (23 OTUs), and *Lokiarchaeota* (1 OTU). The first two of these were the most abundant, accounting for 98.8% of the archaeal community. At class and family levels, Marine Group I (MG-I) of *Thaumarchaeota* and MG-II of *Euryarchaeota* were the predominant groups constituting 62.3% and 33.6% of the whole archaeal community, respectively. This was followed by *Euryarchaeota* MG-III which comprised 2.43% of the total.

A total of 33 OTUs were affiliated with *Thaumarchaeota*, which included 24 MG-I OTUs and 9 Soil Crenarchaeotic Group (SCG) OTUs. The MG-I OTUs accounted for 99.7% of all thaumarchaeotal sequences, and were mainly represented by two phylotypes i.e., *Nitrosopelagicus*-like (ten OTUs) and *Nitrosopumilus*-like (three OTUs) lineages (Supplementary Fig. S1 and Table S2). These comprised 68.3% and 29.8% of the MG-I community, respectively. Also, the WCB group was present, accounting for 0.5% of the community (eight OTUs). Of the other three MG-I OTUs, one was affiliated with *Nitrosarchaeum* whereas the other two did not have any confirmed classifications. Overall, the *Nitrosopelagicus*-like phylotype was the most abundant thaumarchaeotal clade in the eastern Chinese marginal seas.

### Spatial distribution of *Thaumarchaeota* in the surface and SML

**Fig. 3:**
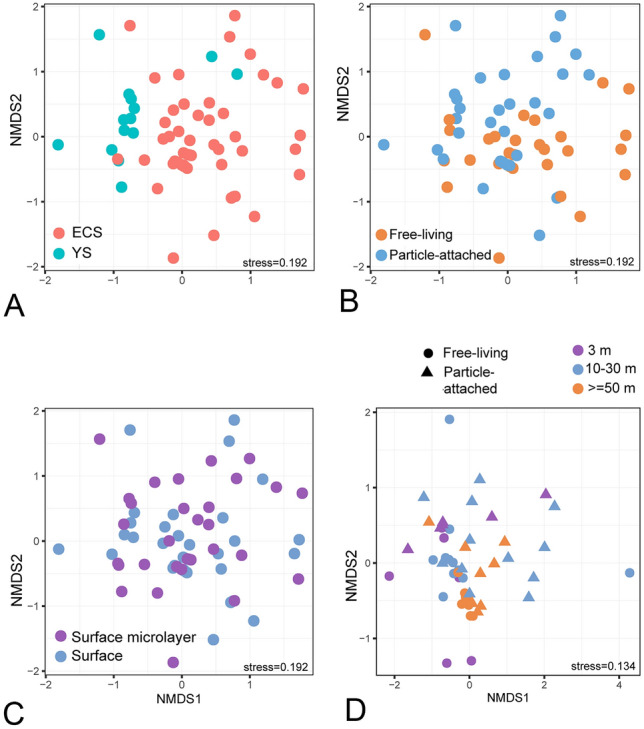
Non-metric multidimensional scaling plots of the thaumarchaeotal communities. **A**, **B** comparison of surface and surface microlayer between sampling areas and lifestyles. **C** comparison between surface and surface microlayer. **D** comparison between different water depths of ECS sites

The surface water (from 20 sites) and SML (from 17 sites) samples were separately analyzed to explore thaumarchaeotal horizontal distribution patterns. The OTU-level non-metric multidimensional scaling analysis (NMDS) plot revealed a significant community separation based on sampling area, with the ECS and YS communities forming two geographic clusters (Fig. 3A; PERMANOVA, *R*^2^ = 0.085, *P* = 0.001). This spatial community segregation was significant in both the surface water (*R*^2^ = 0.122, *P* = 0.001) and SML (*R*^2^ = 0.088, *P* = 0.017) (Supplementary Fig. S2). By comparison, no significant separations were observed between lifestyles (free-living *verse* particle-attached fractions) according to the PERMANOVA analysis (*R*^2^ = 0.017, *P* = 0.303), consistent with the NMDS analysis showing overlap of samples from the two fractions (Fig. 3B). Both the free-living and particle-attached communities displayed spatial segregation between the YS and ECS, and had significant correlations with temperature (Supplementary Table S3) and PCNM 1 (the first component of the principal coordinates of neighborhood matrix; Supplementary Fig. S3) as shown by the distance-based redundancy analysis (db-RDA). Overall, *Thaumarchaeota* communities in surface water and SML were more variable between areas than between lifestyles.

Despite the overall community similarity of free-living and particle-attached *Thaumarchaeota*, calculation of Bray–Curtis dissimilarity showed a higher degree of lifestyle partitioning in surface waters (Bray–Curtis dissimilarities averaged 0.82 ± 0.21) than in SML (0.76 ± 0.22; Welch *t* test, *P* < 0.001). Moreover, it was found that these two fractions displayed contrasting horizontal distribution patterns between surface water and SML (Supplementary Fig. S4). In surface waters, both the fractions varied significantly between the YS and ECS (*R*^2^ = 0.180, *P* = 0.002 and *R*^2^ = 0.150, *P* = 0.002 for free-living and particle-attached, respectively). In SML, the YS–ECS community partition was observed only in the free-living fraction (*R*^2^ = 0.132, *P* = 0.038) but not in its particle-attached counterpart (*R*^2^ = 0.065, *P* = 0.537). This meant that particle-attached *Thaumarchaeota* in SML are more similar independent of the geographical location.

**Fig. 4:**
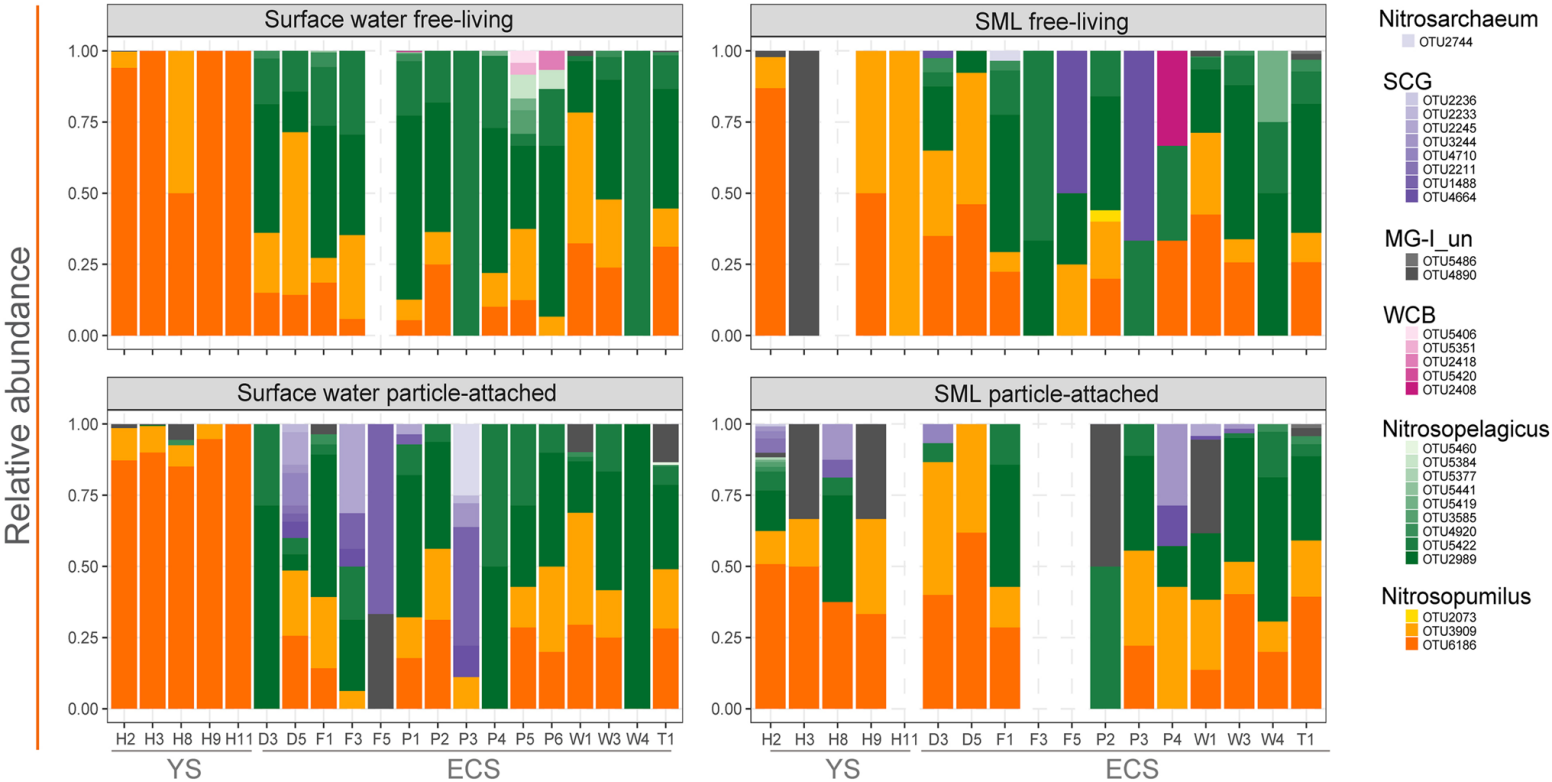
Community composition of *Thaumarchaeota* between free-living and particle-attached fractions in the surface water and surface microlayer (SML). OTU classification was inferred according to both phylogeny and comparison to the Silva database (Supplementary Fig. S2). The clustering relationship of OTU 4890 and 5486 was uncertain and they are shown as MG-I unclassified (MGI_un). The blank areas indicate no *Thaumarchaeota* OTUs in the total prokaryotic community. *YS* Yellow Sea; *ECS* East China Sea

The observed spatial heterogeneity in the surface water and SML was mirrored by variations in the relative abundance of dominant lineages between areas (Fig. 4; Supplementary Fig. S5). Nearly all of the thaumarchaeotal members in the YS belonged to the *Nitrosopumilus*-like phylotype, and a single OTU constituted > 80% of the community at most YS sites. By contrast, the ECS sites were populated by a more diverse community represented mainly by the *Nitrosopelagicus*-like phylotype. Also, the SCG clade had a high abundance in ECS, particularly at sites near the Changjiang estuary, indicating a potential influence of river discharge. The relative distribution of the dominant thaumarchaeotal groups showed similar dynamic trends between the surface water and SML (Supplementary Fig. S5). However, for SML, the particle-attached community in YS, except for H11, appeared to be more diverse than in the corresponding surface water (Fig. 4). This observation was supported by comparison using Shannon diversity (Welch *t*-test, *P* = 0.005; Supplementary Fig. S6). By contrast, no similar results were observed in the ECS. Spatial heterogeneity of particle-attached *Thaumarchaeota* was lower in the SML as shown above. In the ECS, the increase of the SCG clade in surface water was mainly reflected in the particle-attached fraction.

### Vertical distribution of *Thaumarchaeota*

**Fig. 5:**
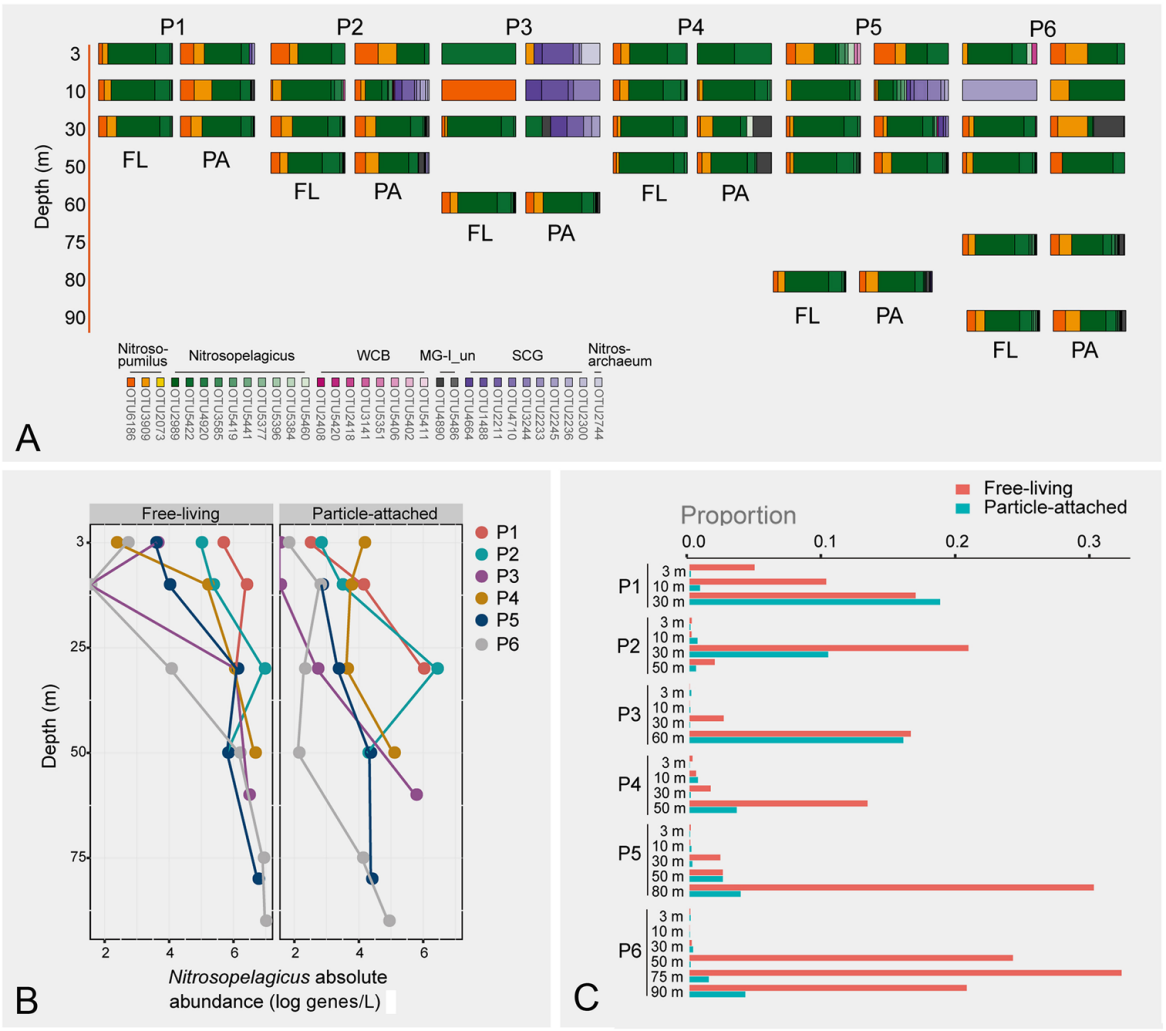
*Thaumarchaeota* community at different water depths of ECS sites P1–P6. **A** relative abundance of thaumarchaeotal OTUs; **B** inferred absolute abundance of *Nitrosopelagicus*-like lineage; **C** relative proportion of *Thaumarchaeota* sequence reads in relation to the total number of prokaryotic sequences. *FL* free-living, *PA* particle-attached

Profiling of *Thaumarchaeota* at different depths of the studied area was achieved by a comparison between surface water and SML, and between surface water and lower depths (10–90 m from the ECS sites P1–P6). It was found that there was no clear compositional difference between the surface water and SML across all sites (Fig. 3C). By contrast, a significant community separation between surface water (3 m) and lower depths (10–90 m) was observed (*R*^2^ = 0.096, *P* = 0.002); the surface water was similar to middle water layers (10–30 m; *R*^2^ = 0.074, *P* = 0.051) but was separated from deeper water (≥ 50 m; *R*^2^ = 0.270, *P* = 0.003; Fig. 3D). The *Nitrosopelagicus*-like lineage had the highest relative abundance among other phylotypes at almost all sampling depths (Fig. 5A). Nevertheless, the inferred absolute abundance of *Nitrosopelagicus* (qPCR-derived total *Thaumarchaeota* abundance × relative proportion of *Nitrosopelagicus* in the community) increased with increasing depth (Fig. 5B) and was significantly higher at the greatest depth compared to the surface (Welch *t*-test, *P* < 0.001). This increased abundance of *Nitrosopelagicus* at lower depths may have resulted in an elevated proportion of *Thaumarchaeota* relative to total prokaryotes (Fig. 5C). SCG occupied the 10-m layer at four sites as well as the surface water of site P3 with the highest relative abundance greater than 90% (Fig. 5A).

Both the free-living (*R*^2^ = 0.207, *P* = 0.005) and particle-attached (*R*^2^ = 0.201, *P* = 0.002) *Thaumarchaeota* displayed clear vertical variations in community composition. The latter correlated with dissolved oxygen and ammonium ions whereas the former did not exhibit any correlations with measured environmental variables. Significant community partitioning was found between these two fractions across depths of the P section in the ANOSIM analysis (*R* = 0.062, *P* = 0.029), although not in the PERMANOVA analysis (*R*^2^ = 0.038, *P* = 0.066). Indeed, the Bray–Curtis dissimilarities between free-living and particle-attached fractions (0.81 ± 0.24) were lower than those between surface water and greater depths (0.86 ± 0.20; Welch *t*-test, *P* < 0.001). This indicated that the thaumarchaeotal community was more variable with depth than between size fractions (Fig. 3D).

The lifestyle-based partitioning was more evident at lower depths (10–90 m; 0.79 ± 0.26) than at the surface (0.70 ± 0.26) according to the Bray–Curtis dissimilarity index (Welch *t*-test, *P* < 0.001). This was reflected in a higher relative abundance of the *Nitrosopelagicus*-like phylotype in the free-living fraction than its particle-attached counterpart (Welch *t*-test, *P* = 0.002). The results showed that the free-living community was less diverse compared to that attached to particles (Supplementary Fig. S7). In fact, the presence of SCG OTUs at below-surface depths was mainly associated with particles (Fig. 5A). Free-living *Thaumarchaeota*, although less diverse, was the main contributors to the above-mentioned increase in the proportion of *Thaumarchaeota* relative to total prokaryotes with depth (Fig. 5C).

### Co-occurrence relationship involving *Thaumarchaeota*

**Fig. 6:**
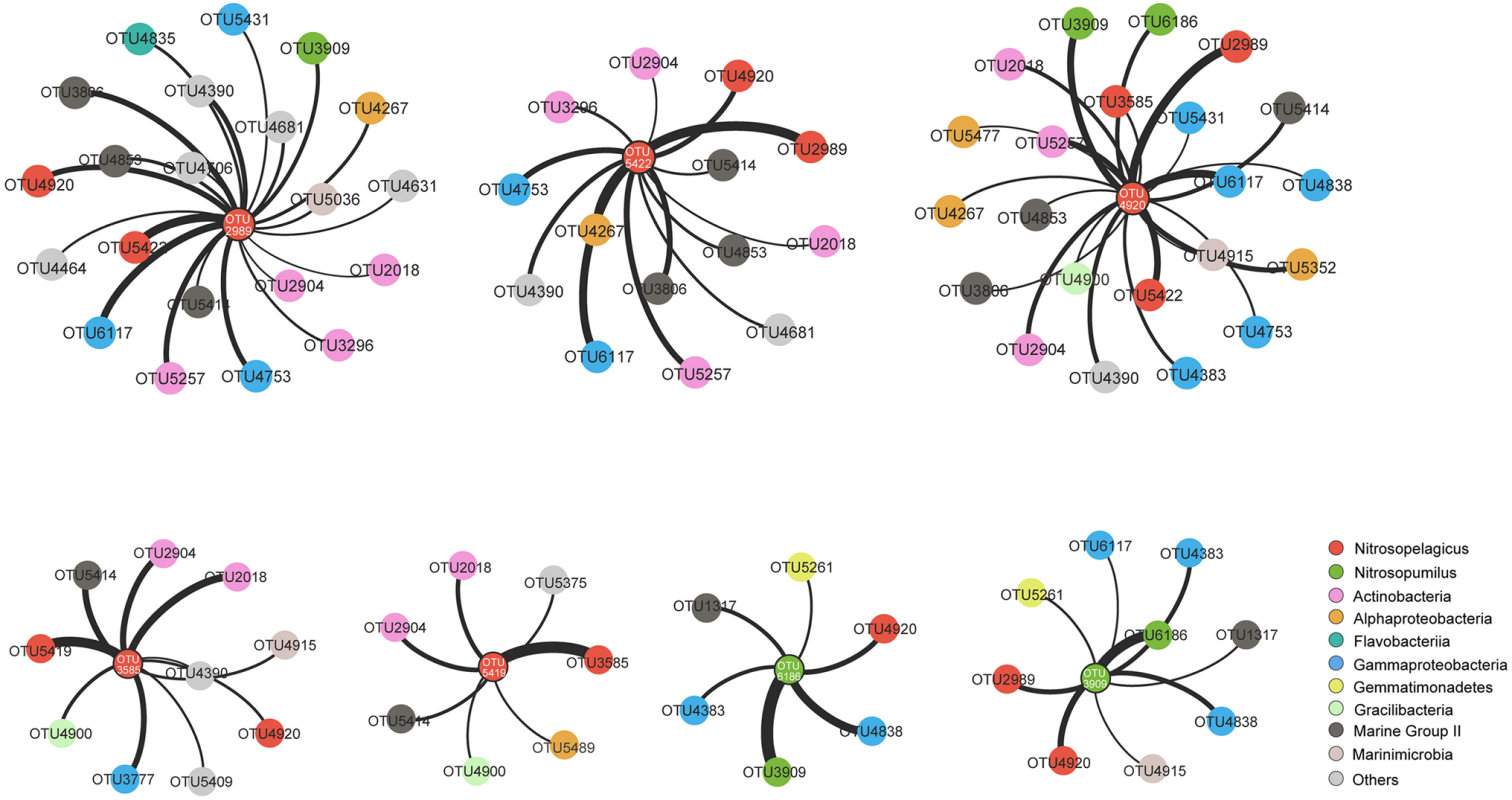
Co-occurrence networks for each of the seven *Thaumarchaeota* OTUs (indicated by red background and white font) involved in the community network. The size of each node is proportional to the number of connections. The thickness of each edge is proportional to the correlation coefficients. All the correlations were positive. All sequence data were included in the network analysis. OTU2989, OTU5422, OTU4920, OTU3585 and OTU5419 were affiliated to *Nitrosopelagicus*; OTU6186 and OTU3909 were affiliated to *Nitrosopumilus*

To explore the potential interactions between thaumarchaeotal OTUs and other microbial taxa, network analyses were performed for 718 bacterial and archaeal OTUs that had an average abundance of > 0.01% across all samples. The thaumarchaeotal community involved in the network was represented by seven OTUs, which showed correlations to a wide taxonomical range, including *Gammaproteobacteria*, *Actinobacteria*, *Alphaproteobacteria*, *Marinimicrobia* and euryarchaeotal MG-II (Fig. 6). Of the seven OTUs, five and two were affiliated with *Nitrosopelagicus*-like and *Nitrosopumilus*-like phylotypes, respectively. Correlations between the two phylotypes and/or within members of a single phylotype were observed. Both the two phylotypes showed co-occurrence links with MG-II and *Gammaproteobacteria*; however, within *Gammaproteobacteria*, the E01-9C-26 group was only correlated with the *Nitrosopelagicus*-like phylotype, whereas the *Nitrosopumilus*-like phylotype was more correlated with ZD0405 and JTB255. Moreover, links with *Actinobacteria* (mainly clade Sva0996) and *Alphaproteobacteria* (mainly clade PS1) were exclusively found in the *Nitrosopelagicus*-like phylotype. In contrast, correlations with *Gemmatimonadetes* were only observed in the *Nitrosopumilus*-like phylotype (Fig. 6).

The effect of size fractions on the co-occurrence pattern was then examined by distinguishing the free-living and particle-attached fractions. With an average abundance of > 0.01%, 546 and 859 OTUs for free-living and particle-attached fractions, respectively, were selected for the analysis. Although a lower number of OTUs was used for the free-living fraction, 9 thaumarchaeotal OTUs were involved in the network, and had 253 connections in total with other microbial taxa. In comparison, 6 thaumarchaeotal OTUs were included in the network of the particle-attached fraction, and revealed only 76 connections, which were substantially lower than in the free-living network. Different *Thaumarchaeota*-microbial taxa co-occurrence relationships were observed between the free-living and particle-attached fractions (Supplementary Fig. S8). For example, OTUs affiliated with *Thermoplasmata* (mainly MG-II), *Marinimicrobia*, and *Alphaproteobacteria* were mainly connected with free-living thaumarchaeotal OTUs, whereas those belonged to *Deltaproteobacteria* and *Planctomycetacia* showed higher connections with particle-attached OTUs.

## Discussion

### Basin-related spatial separation of *Thaumarchaeota* assemblages

The spatial pattern of planktonic *Thaumarchaeota* in the YS and ECS of the Chinese marginal seas was investigated. These two areas have contrasting hydrological conditions with respect to ocean currents, river discharges, and water mass (including Kuroshio) intrusion and have been reported to host different microbial communities especially in the sediment (Liu et al. 2020). Indeed, results presented here show that the YS surface water harbored a thaumarchaeotal community with lower abundance and species diversity compared to the ECS. The thaumarchaeotal community in the YS was characterized by a single OTU affiliated to the *Nitrosopumilus*-like phylotype. Certainly, the predominance of a single species-level OTU of *Thaumarchaeota* has previously been observed in other coastal environments (Muller et al. 2018; Tolar et al. 2020) but this is not expected considering the role of protist grazing and virus lysis in regulating community stability. Indeed, archaeal-specific predation by eukaryotes has been documented in the North Atlantic Basin (Seyler et al. 2019). However, *Nitrosopumilus* spp. have genes related to predator and viral defense (e.g., glycoprotein layer and DNA modification; Ahlgren et al. 2017; Stieglmeier et al. 2014). More importantly, the low proportion of *Thaumarchaeota* in the total prokaryotic community may have weakened the effect of these biological processes (Herber et al. 2020). A possible explanation is high resistance of the *Thaumarchaeota* to environmental disturbance in the YS, which needs further elucidation.

In the ECS, *Thaumarchaeota* was more abundant and shifted to a community composition dominated by the *Nitrosopelagicus*-like phylotype. This suggests that certain environmental conditions may have promoted the proliferation of the *Nitrosopelagicus*-like phylotype in the ECS. In this respect, temperature showed positive correlations with the abundance of the *Nitrosopelagicus*-like lineage. This was in contrast to previous findings which found that a *Nitrosopelagicus* enrichment culture had a lower growth temperature optimum than *Nitrosopumilus maritimus* (Martens-Habbena et al. 2009; Santoro et al. 2015). However, available cultures constitute only a small fraction of the natural thaumarchaeotal species diversity (Alves et al. 2018). Indeed, abundant *Nitrosopumilus*-like sequences have been found in global high-latitude seawater, demonstrating that at least some species prefer low-temperature conditions (Cheung et al. 2019). Although not measured here, the previously reported higher concentration of dissolved organic matter in surface water of the YS relative to the ECS (Yang et al. 2021) may also contribute to the niche partitioning between the two phylotypes. *Thaumarchaeota* is typically autotrophic (Könneke et al. 2005) with the potential of carbon fixation through the modified 3-hydroxypropionate/4-hydroxybutyrate (3-HP/4-HB) cycle (Könneke et al. 2014). Nevertheless, two strains of *Nitrosopumilus* have been found to require α-ketoglutaric acid as the organic source for growth (Qin et al. 2014), although a role for α-ketoglutaric acid as a H_2_O_2_ scavenger was proposed subsequently (Kim et al. 2016). Also, field studies have provided evidence for heterotrophy in *Thaumarchaeota* in both pelagic and benthic marine environments (Biddle et al. 2006; Ouverney and Fuhrman 2000; Seyler et al. 2014, 2018). Thus, the dominance of *Nitrosopumilus*-like phylotype in YS may relate to a high organic matter availability. The discovery of enriched *Nitrosopumilus*-like phylotype in the hadal ocean (> 6000 m water depth) with enhanced organic matter deposition supports this possibility (Zhong et al. 2020). Also, a recent survey of AOA in the global ocean revealed the dominance of *Nitrosopumilus*-like and *Nitrosopelagicus*-like phylotypes in coastal and ocean opens, respectively (Qin et al. 2020). These findings suggest that the *Nitrosopumilus*-like phylotype may adapt to the coastal surface water by adopting a mixotrophic lifestyle.

### Differences in *Thaumarchaeota* communities between surface water and SML

SML is physico-chemically different from the underlying surface water by being enriched in organic and inorganic nutrients, and thereby microbial cells (Cunliffe et al. 2011). In this study, no significant difference in thaumarchaeotal abundance was observed between surface water and SML. This is in contrary to previous findings of higher bacterial abundance in the SML than in surface water (Aller et al. 2005; Sun et al. 2020). Interestingly, Auguet and Casamayor (2008) found that in some high mountain lakes, there was an enrichment of archaea in the SML relative to the underlying water. Compared to lakes, the hydrodynamic environments in coastal waters are more complex, and physical disturbances occur frequently. This may create unstable SML environments preventing the enrichment of archaea. As a consequence, the thaumarchaeotal community compositions were similar in SML and surface water. Also, little effect of SML on archaeal communities has been observed in other coastal sites (Cunliffe et al. 2009; Wong et al. 2018), suggesting that the SML environment is less favorable to archaea than to bacteria (Cunliffe et al. 2009).

Despite the overall community homogeneity, *Thaumarchaeota* in the SML and surface water displayed different spatial distribution patterns and lifestyle partitioning. Free-living *Thaumarchaeota* was separated by water body in both SML and surface water. By contrast, the particle-attached fraction exhibited spatial segregation only in surface water but not in SML. This indicates the specific effects of SML on the distribution of particle-attached *Thaumarchaeota*. Particles are frequently enriched in SML, which serve as the basis for the formation of marine aerosols (Aller et al. 2005). Aerosolization releases microbial cells trapped in the organic particles of SML into the atmosphere, acting as a dispersal mechanism for marine microorganisms (Aller et al. 2005; Engel et al. 2017). Thus, the spatial homogeneity of particle-attached *Thaumarchaeota* in SML may result from the high dispersal rates. This scenario is different from that commonly seen in water where the particle-attached community is more constrained by dispersal limiting than its free-living counterpart (Liu et al. 2019b; Wang et al. 2020). Results presented here highlight contrasting microbial biogeography of size-fractionated samples between SML and surface water.

### Size-fractionated niche partitioning was magnified at lower depths (10–90 m)

A clear increase in *Thaumarchaeota* abundance and proportion relative to prokaryotes from surface to bottom water (< 100 m) was observed at the ECS sites. Similarly, a study by Wang et al. (2019) reported the maximum concentration of archaeal isoprenoid glycerol dialkyl glycerol tetraethers at shallow ECS sites (< 100 m). This elevated *Thaumarchaeota* abundance with depth was mirrored by a significant increase in the abundance of the *Nitrosopelagicus*-like phylotype. This scenario is different from the previously reported vertical partitioning of *Thaumarchaeota* between the sunlit surface ocean and the aphotic deep ocean, where WCB is the most prevalent clade (Francis et al. 2005; Sintes et al. 2013; Smith et al. 2014a). Thus, evidence for a vertical variation of *Nitrosopelagicus* in the shallow shelf region is provided here, which suggests the existence of a specific ecological niche for the *Nitrosopelagicus*-like phylotype in shallow subsurface waters. However, these aspects may include a variety of environmental forces, including temperature, dissolved oxygen, and light. These have been found to influence the distribution and physiological activity of other thaumarchaeotal species (Hugoni et al. 2013; Qin et al. 2014, 2017).

It is notable that the increase in abundance of the *Nitrosopelagicus*-like phylotype at greater depths in the ECS, occurred mainly in the free-living lifestyle, resulting in elevated proportion of *Thaumarchaeota* in the free-living prokaryotic communities. This was consistent with previous findings that *Thaumarchaeota* prefers to adopt a free-living lifestyle (Smith et al. 2013; Zhong et al. 2020)*.* In contrast, in surface water and SML of the YS dominated by *Nitrosopumilus*-like sequences, no differences in abundance and community composition were observed between free-living and particle-attached fractions. It is likely that at least some members of *Nitrosopumilus* persisting in the surface water had evolved the ability to adhere to particles. Supporting this, Bayer et al. (2016) suggested a particle-attached life mode in *Nitrosopumilus adriaticus* due to the potential capability in seeking favorable microhabitats with concentrated particles. Also, the association of *Nitrosopumilus* with particles has been observed in other estuaries and coastal seas (Li et al. 2018; Wäge et al. 2019). Indeed, the first isolate of *Nitrosopumilus* was purified from an enrichment with marine tank gravels (Könneke et al. 2005). These findings suggest different life modes between *Nitrosopelagicus* and *Nitrosopumilus,* with the latter preferring to attach to particulates, probably as a survival strategy for absorbing organic matter (Qin et al. 2014) and/or avoiding sunlight (Horak et al. 2018). The differential presumed lifestyle and distribution pattern between the two phylotypes led to enhanced size-fractioned niche partitioning of *Thaumarchaeota* in the shallow subsurface water compared to the surface water.

### Co-occurrence pattern varied between phylotypes and lifestyles

To a certain extent, microbial distributions may be explained by taxa–taxa interactions (Liu et al. 2019a). Using correlation-based networks, a wide co-occurrence relationship was found between *Thaumarchaeota* and other bacterial and archaeal taxa. Common associations among different phylotypes included positive correlations to members of *Marinimicrobia* and archaeal MG-II. Potential N_2_O-involved metabolic coupling between *Thaumarchaeota* and *Marinimicrobia*, with the capacity in producing and reducing N_2_O, respectively, has been inferred via omic technologies (Hawley et al. 2017), and indicated by network analyses (Reji et al. 2019). A combination of results from Reji et al. (2019) and the present study suggest that associations of *Thaumarchaeota* and *Marinimicrobia* may be present in both epipelagic and mesopelagic depths. MG-II is a heterotrophic archaeal group dominating the surface ocean (Zhang et al. 2015); its link to *Thaumarchaeota* persisting in the euphotic zone may indicate metabolic cross-feeding since their representatives have different trophic modes. However, caution is needed when interpreting these results as co-occurrence patterns do not always represent true interactions, but may just indicate similar habitat preferences (Liu et al. 2019a).

Similar to the results of Reji et al. (2019), varying co-occurrence relationships were seen between *Nitrosopumilus*-like and *Nitrosopelagicus*-like phylotypes. For example, correlations to *Gemmatimonadetes* were only seen in the *Nitrosopumilus*-like lineage. Codominance of *Gemmatimonadetes* and thaumarchaeotal MG-I has been observed in oxic deep-sea sediments (Durbin and Teske 2011). Certainly, the genomic potential for protein degradation in *Gemmatimonadetes* (Baker et al. 2015) may provide ammonia for *Nitrosopumilus*. Indeed, *Nitrosopumilus* is the dominant benthic thaumarchaeotal group rather than *Nitrosopelagicus* (Alves et al. 2018). The *Nitrosopumilus*-like phylotype also showed correlations with other heterotrophic taxa such as gammaproteobacterial ZD0405, which has been reported dominant during algae blooms (Teeling et al. 2016). These results may indicate the nutritional reliance of *Nitrosopumilus* on microorganisms capable of degrading complex organic matter. Metabolic associations may also occur in the *Nitrosopelagicus*-like phylotype, but with different partners and mechanisms. For example, utilization of simplified organic matter (such as C1 and amine compound) by gammaproteobacterial E01-9C-26 group (Landry et al. 2018) may provide substrate for *Nitrosopelagicus.* The specific correlation of the *Nitrosopelagicus*-like phylotype to Sva0996 may indicate habitat preference, as both of them increased in abundance below the surface (Reintjes et al. 2019). Additionally, there were variations in co-occurrence patterns between size fractions. Notably, the free-living *Thaumarchaeota* showed higher connectivity to other taxa than the particle-attached members. This may suggest the occurrence of broad inter-species interactions in free-living *Thaumarchaeota*, potentially serving as a survival strategy for nutrient exchange (Doxey et al. 2015). Possibly, this may differ from the particle-attached fraction in which the correlations to *Deltaproteobacteria* and *Planctomycetacia* preferring to attach to particles (Mestre et al. 2017) may reflect their similar environmental niches. A significant correlation was observed between free-living *Thaumarchaeota* and nitrite-oxidizing bacteria affiliated with *Nitrospinia*, suggesting that the ammonia-oxidizing activity of *Thaumarchaeota* is more likely to occur when cells are free living rather than attached to particles. Our findings of phylotype and lifestyle-specific co-occurrence patterns highlight the importance of *Thaumarchaeota* analyses at a finer taxonomic level.

## Conclusion

This study has demonstrated horizontal and vertical heterogeneity in the community structure of planktonic *Thaumarchaeota* in the shallow shelf region of the Chinese marginal seas. *Thaumarchaeota* in SML and surface water were similar in community composition, but exhibited different spatial patterns, with the particle-attached fractions being more easily dispersed in SML than surface water. Depth-related increase in *Thaumarchaeota* abundance and change in lifestyle were observed. Size-fractionated niche partitioning was more pronounced at shallow subsurface water than in surface water, which may be attributed to varying life modes between *Nitrosopelagicus* and *Nitrosopumilus*. Attachment to particles may act as a survival strategy for certain *Nitrosopumilus*-like species inhabiting the surface ocean. Changes of lifestyle may be promoted by specific correlations between *Thaumarchaeota* phylotypes and other microorganisms. This study provides useful insights into the thaumarchaeotal community distribution in the coastal surface ocean at a finer phylotype level, and raises important questions that warrant further investigation, such as how different *Thaumarchaeota* phylotypes coexist and diverge in the surface ocean, and which phylotype plays a more important role in the ammonia oxidation.

## Materials and methods

### Sampling

Seawater samples were collected at 26 sites along the eastern Chinese marginal seas aboard the R/V *Dong Fang Hong 2* from June to July 2018 (Fig. 1). Ten sites were located in the YS, whereas the other sixteen were located in the ECS. Samples were collected from different depths (from surface to the near bottom) at P1–P6 sites of ECS, and only surface water was collected at other sites (Supplementary Table S1). In addition, 17 sites (Supplementary Table S1) were collected for SML samples using a Garrett metal screen (MS) as previously described, with the depth of the SML samples being ~ 300–1000 µm (Yang et al. 2022). To compare community composition between size fractions, all samples except for the surface water of H1, H10, B1, B3, B5, and T3 were separated into particle-attached (> 3 μm) and free-living (0.22–3 μm) communities. Specifically, 300 mL of SML samples and 1000 mL of samples at each other depth were filtered serially through 3-μm-pore-size and 0.22-μm-pore-size polycarbonate membranes (Millipore Corporation, Billerica, MA) using a vacuum pump at a pressure of < 0.03 MPa on board the ship. The result was 120 samples, in total. After cell collection, the filters were stored at -20 °C onboard, and -80 °C in the laboratory. Seawater salinity, temperature, and depth were recorded by a Seabird 911-plus conductivity–temperature–depth (CTD) system (Sea-Bird Electronics Inc., Bellevue, WA, USA). Dissolved inorganic nutrients (NO_3_^−^, NO_2_^−^, NH_4_^+^, SiO_4_^2−^ and PO_4_^3−^) were analyzed for the surface water and samples from different depths at ECS sites P1–P6 using an AA3 autoanalyzer system (Seal Analytical Ltd., Southampton, UK).

### DNA extraction, sequencing, and quantitative PCR

Total DNA was extracted from filters using the PowerSoil DNA Isolation Kit as previously described by Sun et al. (2020). 515F (5′-GTGYCAGCMGCCGCGGTAA-3′) and 806R (5′-GGACTACNVGGGTWTCTAAT-3′) primers (Apprill et al. 2015; Parada et al. 2016) were used for prokaryotic 16S rRNA gene amplification. The reactions occurred in a 20-μL system containing 0.4 μL of Fastpfu polymerase, 4 μL of FastPfu Buffer (5 ×), 2 μL of dNTP mix (2.5 mmol/L), 0.8 μL of each primer (5 μmol/L), and 10 ng of template DNA. The thermal conditions were 95 °C for 3 min, 29 cycles of 95 °C for 30 s, 55 °C for 30 s, 72 °C for 45 s, and a final extension at 72 °C for 10 min. Triplicate amplifications were conducted for each sample, and were mixed for library preparation. Pair end sequencing was carried out by the Majorbio Bio-Pharm Technology, Shanghai, China with the Illumina Miseq platform. The raw reads have been deposited in the NCBI SRA database under the accession number PRJNA648032.

*Thaumarchaeota* abundance was measured using quantitative PCR (qPCR) with the primers GI334F (5′-AGATGGGTACTGAGACACGGAC-3′) and GI554R (5′-CTGTAGGCCCAATAATCATCCT-3′) (Suzuki et al. 2000). The reactions occurred in a 20-μL system containing 10 µL SYBR Premix Ex Taq II (2 ×), 0.4 µL ROX Reference Dye II (50 ×), 0.8 µL each primer, and 2 µL DNA template. The thermal conditions were 94 °C for 2 min, followed by 40 cycles of 94 °C for 15 s, 59 °C for 60 s, and 72 °C for 30 s. Triplicate amplifications were performed for each sample. Tenfold serially diluted linear plasmids containing a single copy of the thaumarchaeotal 16S rRNA gene (the sequence was amplified from a coastal water sample and shown in the supplementary material) were used to generate the standard curve. The amplification efficiency varied between 0.94 and 0.99, and *R*^2^ was greater than 0.999.

### OTU clustering and statistical analysis

Raw reads were quality controlled using Trimmomatic (Bolger et al. 2014) following the criteria as previously reported (Liu et al. 2020). FLASH (Magoc and Salzberg 2011) was used to merge the pair end reads. Operational taxonomic units (OTUs) were clustered in UPARSE (Edgar 2013) at a 97% similarity cutoff and taxonomically assigned using the RDP classifier against the Silva database Release 128 (http://www.arb-silva.de). Sequencing depth of each sample was equalized to 28,134 reads corresponding to the lowest sequence number among all samples. The *Thaumarchaeota* OTUs were extracted according to the Silva-based taxonomy. Most of the *Thaumarchaeota* OTUs could be annotated at the genus level against the Silva database. Taxonomic affiliation of the OTUs was further confirmed by constructing a neighbor-joining phylogenetic tree using OTU representative sequences and closest neighbors retrieved from the NCBI. To define WCB OTUs, reference sequences reported previously were included (Tolar et al. 2020). The tree was built using the MEGA software (Kumar et al. 2016) with 278 positions, the Kimura 2-parameter model, and 1000 bootstrap replicates. Comparison of the thaumarchaeotal community across samples was performed using the NMDS. The resulting clustering relationship was examined for significance using PERMANOVA analysis except if otherwise specified. Distance-based redundancy analysis (db-RDA) was used to test the relationship between environmental factors and spatial factors and community variations. The spatial factors were generated using the principal coordinates of neighbor matrices (PCNM) analysis. These analyses were conducted with the “vegan” package in R (R Core Team 2015).

Interactions between *Thaumarchaeota* and other microbial taxa were illustrated by the network analysis. The correlation-based co-occurrence patterns were constructed in R using the “igraph” and “Hmisc”. OTUs with a proportion lower than 0.01% across all samples were removed in the analysis. A valid correlation was considered if the pairwise Spearman’s correlations had a correlation coefficient >|0.7| and a *P* value < 0.01 (Benjamini and Hochberg adjusted). The network was visualized in Gephi (Bastian et al. 2009).

## Supplementary Information

Below is the link to the electronic supplementary material.

Supplementary file1 (DOCX 1641 KB)

## Data Availability

The data that support the findings of this study are included in this published article (and its supplementary information file).
