# Supplementary material for: Phylotype resolved spatial variation and association patterns of planktonic Thaumarchaeota in eastern Chinese marginal seas
Source: Mar Life Sci Technol. 2023 Apr 7;5(2):257–70. doi: 10.1007/s42995-023-00169-y (PMC10232715; doi:10.1007/s42995-023-00169-y)
Supplement: Supplementary file 1 — Supplementary file1 (DOCX 1641 KB) [file 42995_2023_169_MOESM1_ESM.docx]

**Supplementary information**

Phylotype resolved spatial variation and association patterns of planktonic *Thaumarchaeota* in the eastern Chinese marginal seas

Jiwen Liu^1,2,3^, Fuyan Huang^1^, Jiao Liu^1^, Xiaoyue Liu^1^, Ruiyun Lin^1^, Xiaosong Zhong^4^, Brian Austin^5^, Xiao-Hua Zhang^1,2,3*^ ([xhzhang@ouc.edu.cn](mailto:xhzhang@ouc.edu.cn))

^1^Frontiers Science Center for Deep Ocean Multispheres and Earth System, and College of Marine Life Sciences, Ocean University of China, Qingdao 266100, China.

^2^Laboratory for Marine Ecology and Environmental Science, Laoshan Laboratory, Qingdao 266237, China.

^3^Institute of Evolution and Marine Biodiversity, Ocean University of China, Qingdao 266003, China.

^4^Key Laboratory of Marine Chemistry Theory and Technology, Ministry of Education, Qingdao 266100, China

^5^Institute of Aquaculture, University of Stirling, Stirling FK9 4LA, Scotland, UK


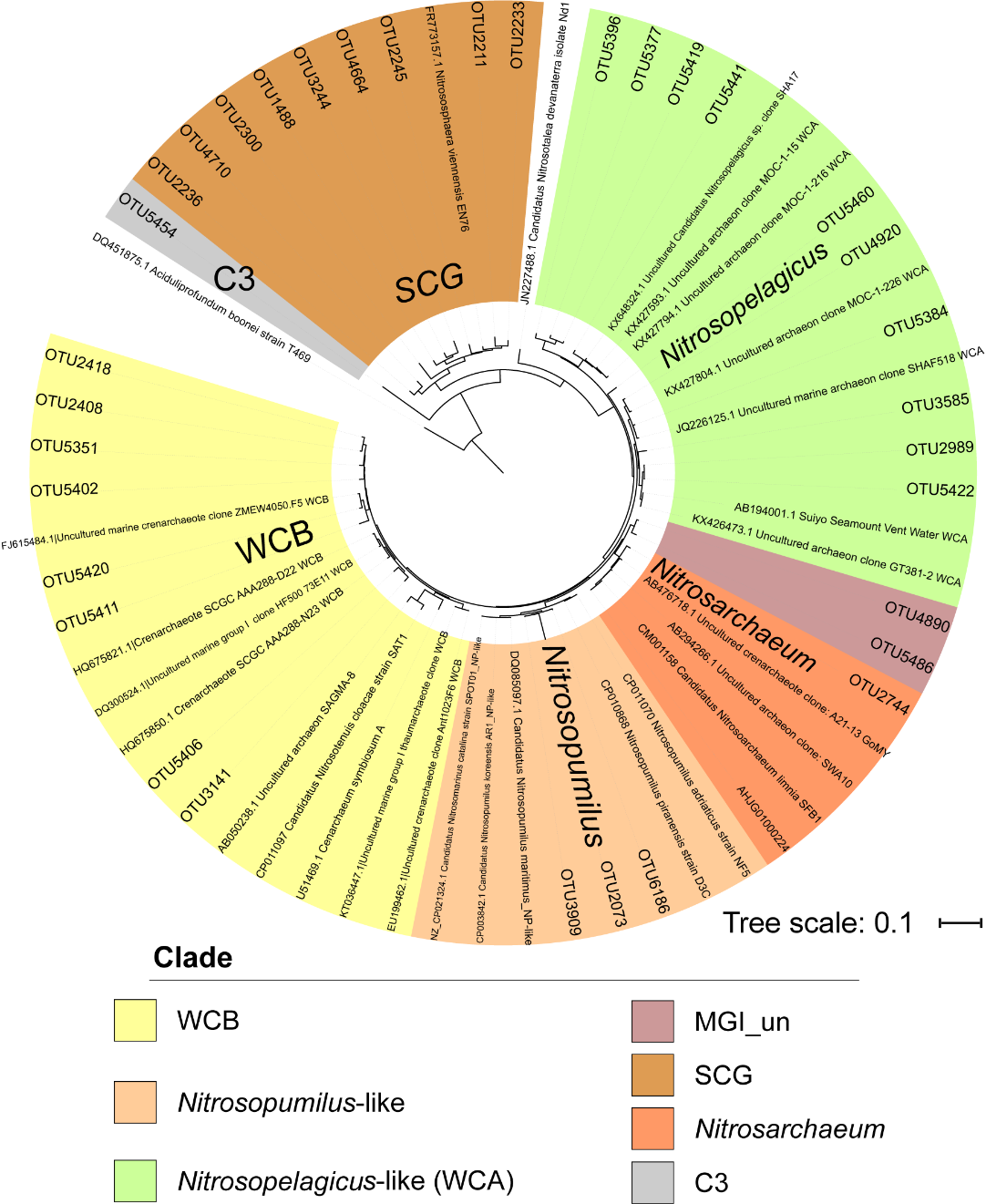


**Supplementary Fig. S1** A neighbor-joining phylogenetic tree constructed using the MEGA software showing the clustering relationship of Marine Group I (MG-I) OTUs. OTU classification was inferred according to both the affiliation in the Silva database and phylogeny. Despite this, the clustering relationship of OTU 4890 and OTU 5486 was uncertain and they were shown as MG-I unclassified (MGI_un).


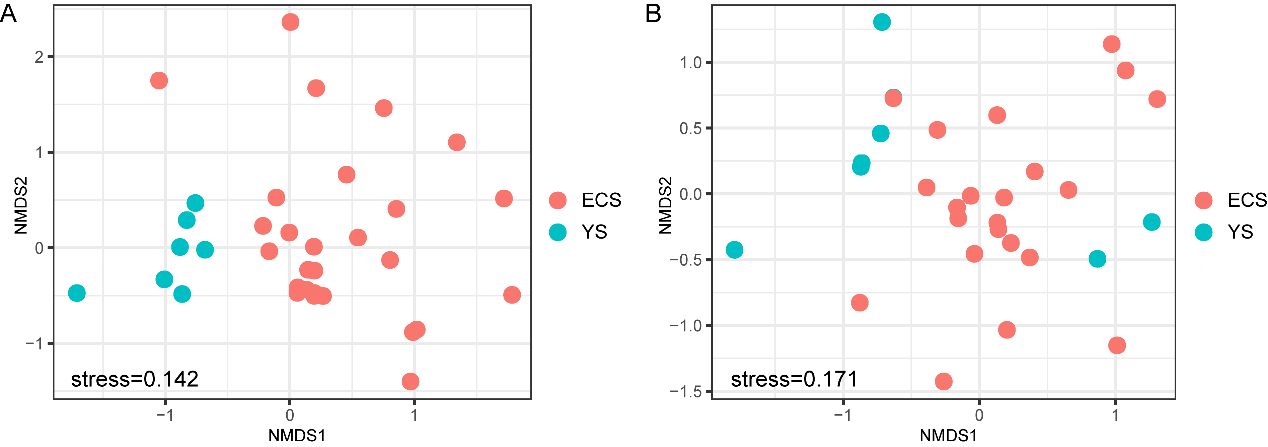


**Supplementary Fig. S2** Non-metric multidimensional scaling analysis of the *Thaumarchaeota* community in surface water (A) and surface microlayer (B) based on the Bray-Curtis dissimilarities. YS, Yellow Sea; ECS, East China Sea.


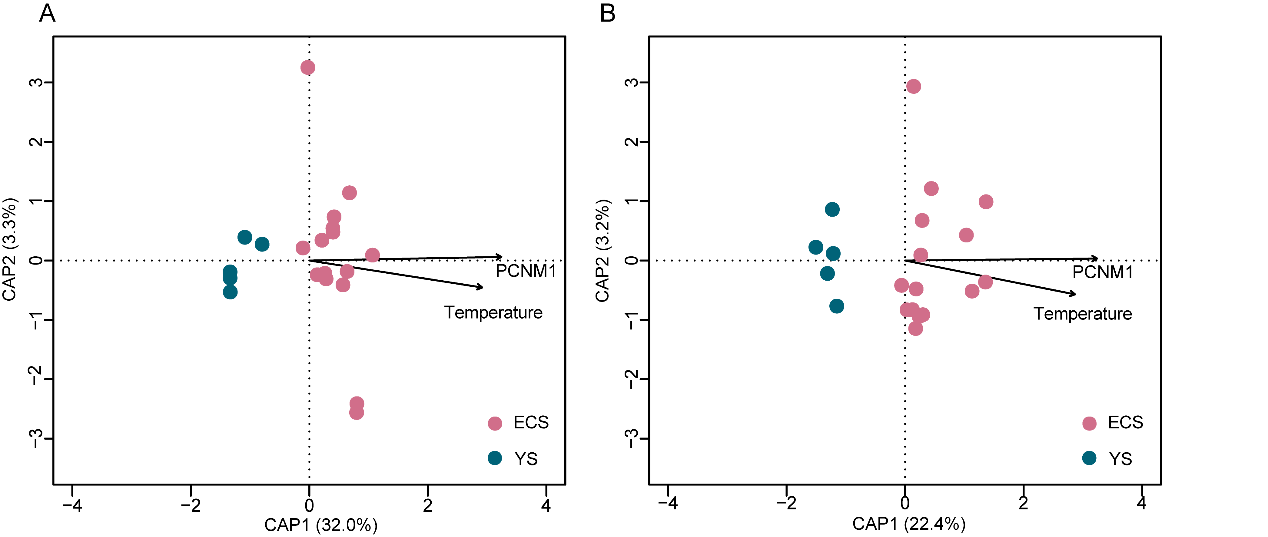


**Supplementary Fig. S3** Distance-based redundancy analysis showing the influence of spatial and environmental factors on the thaumarchaeotal community in the surface water. A, free-living community; B, particle-attached community. Only significant environmental factors are shown on the plot. YS, Yellow Sea; ECS, East China Sea.


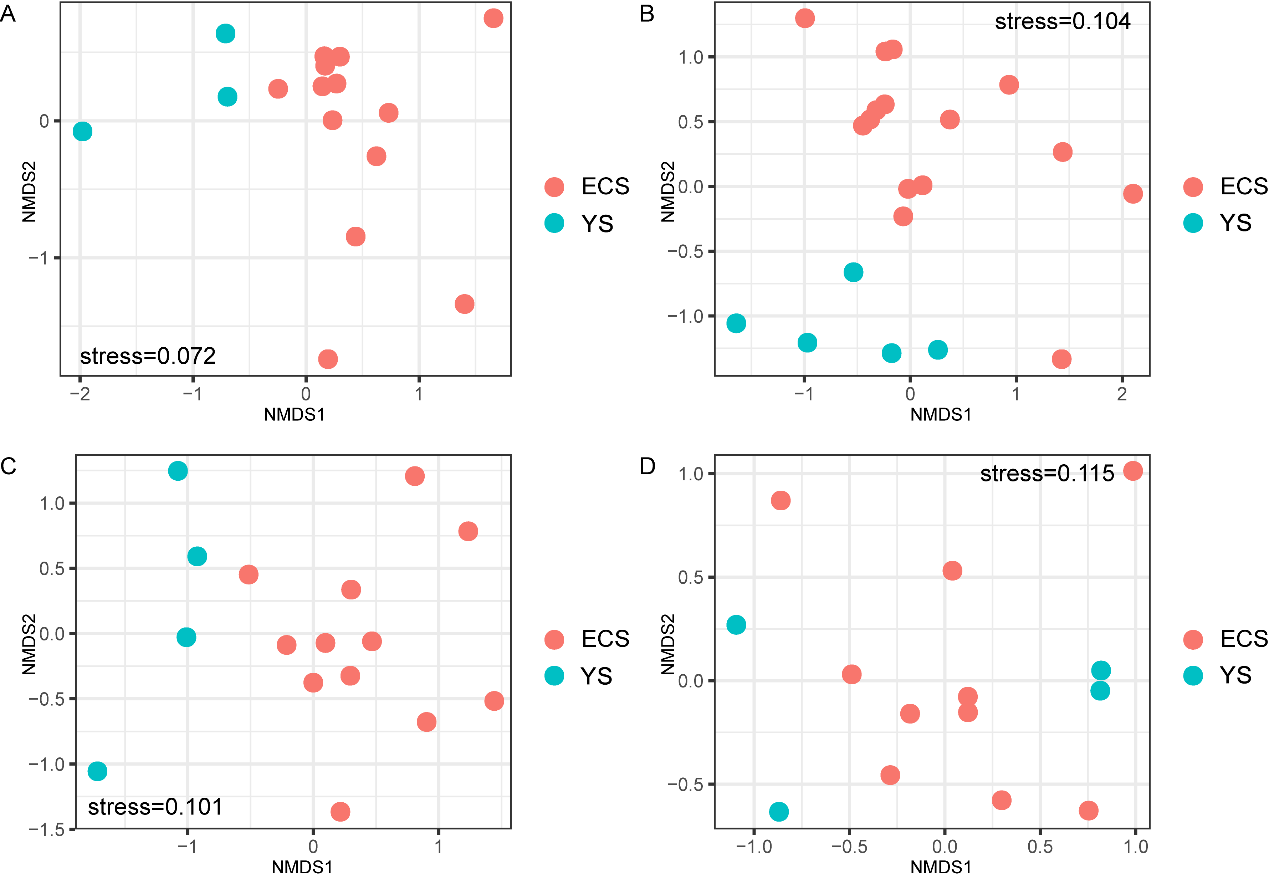


**Supplementary Fig. S4** Non-metric multidimensional scaling analysis of the free-living and particle-attached *Thaumarchaeota* community in surface water and surface microlayer. A, free-living fraction of the surface water; B, particle-attached fraction of the surface water; C, free-living fraction of the micro-surface layer; D, particle-attached fraction of the micro-surface layer. YS, Yellow Sea; ECS, East China Sea.


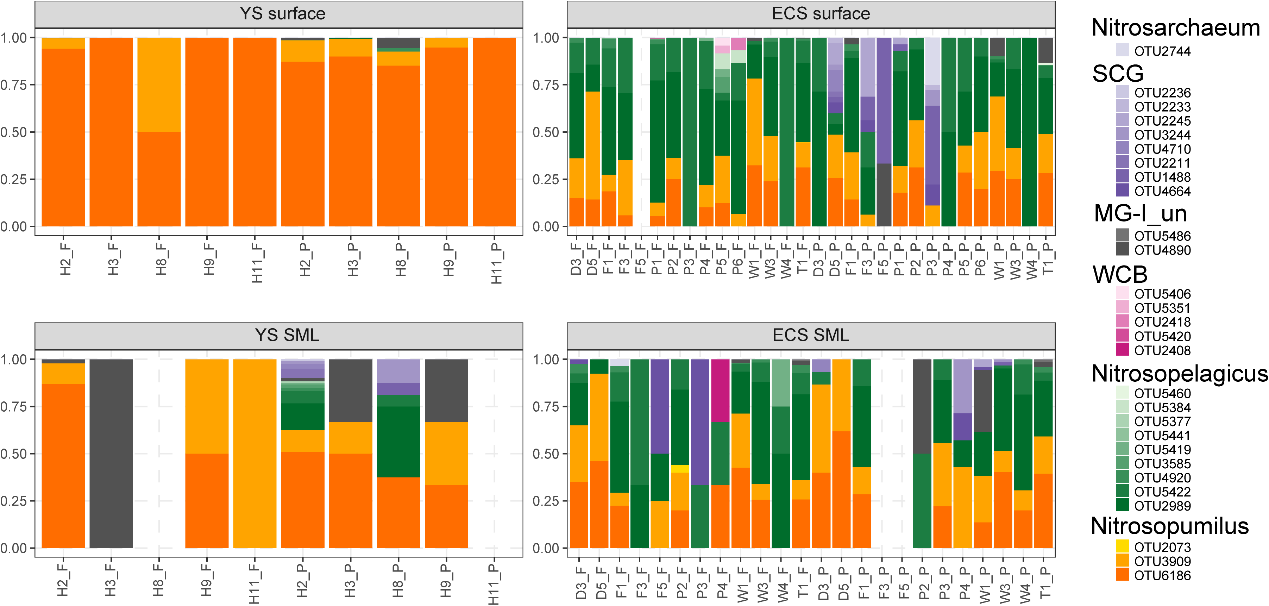


**Supplementary Fig. S5** *Thaumarchaeota* community composition between surface water and surface microlayer (SML) in YS and ECS. YS, Yellow Sea; ECS, East China Sea. The blank areas indicate no *Thaumarchaeota* OTUs in the total prokaryotic community. _P and _F indicate particle-attached and free-living samples, respectively.


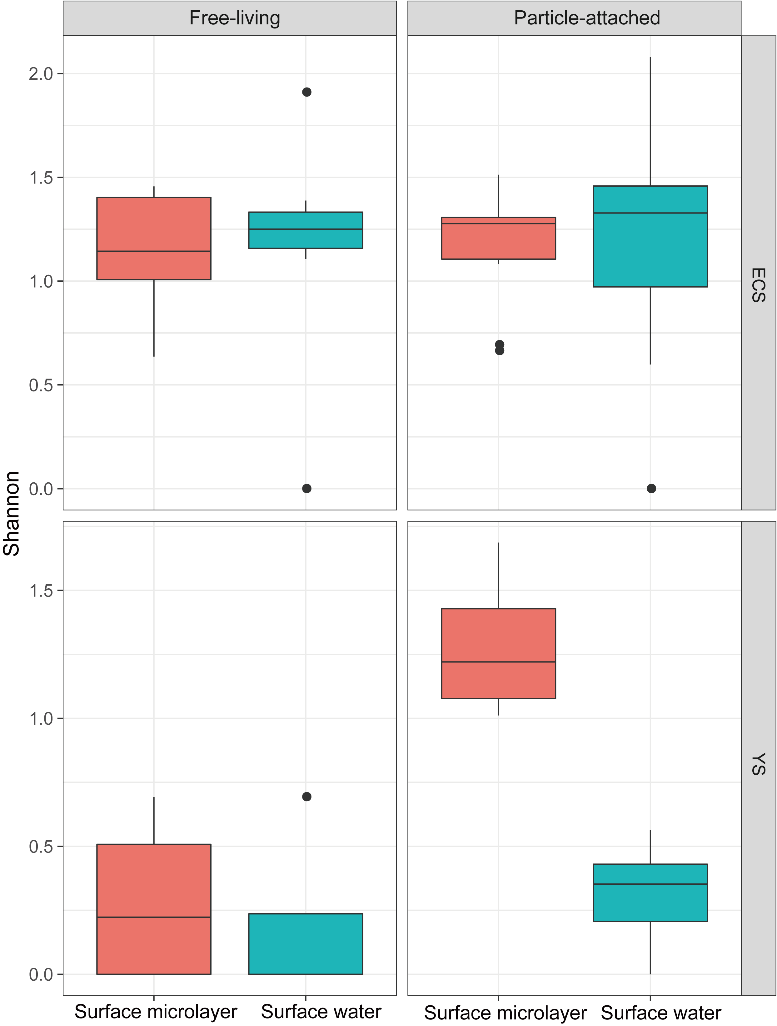


**Supplementary Fig. S6** Comparison of Shannon diversity between free-living and particle-attached *Thaumarchaeota* community in surface microlayer and surface water. YS, Yellow Sea; ECS, East China Sea.


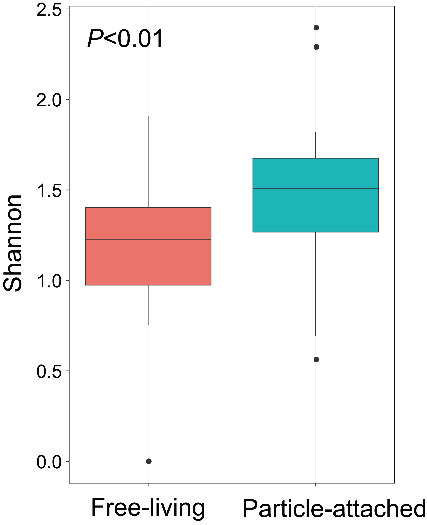


**Supplementary Fig. S7** Comparison of Shannon diversity between free-living and particle-attached *Thaumarchaeota* community across depths of the ECS P section.


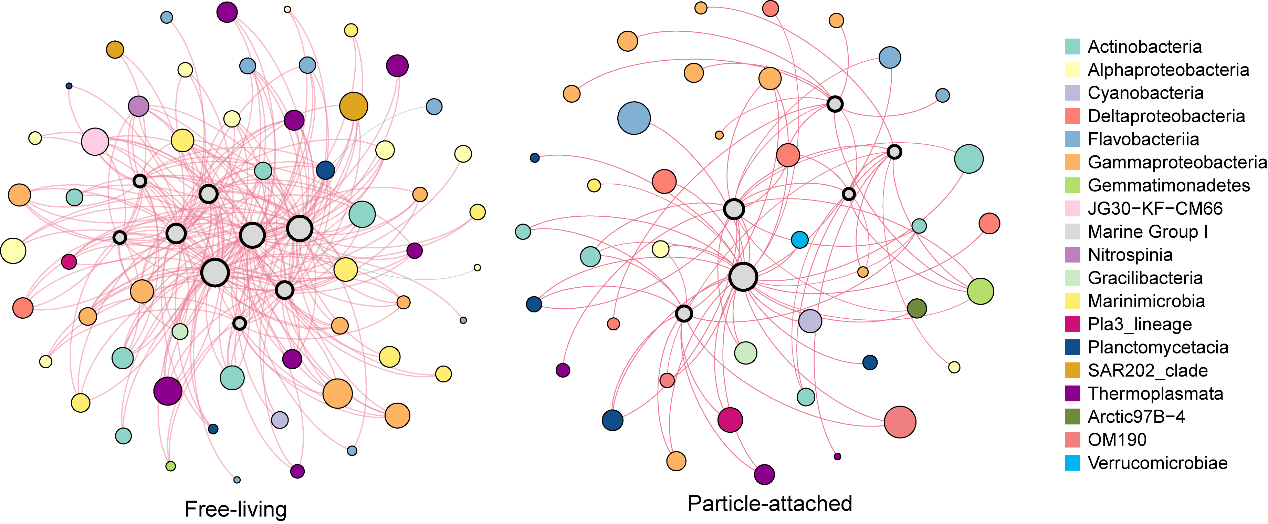


**Supplementary Fig. S8** Network correlation between thaumarchaeotal OTUs (indicated by cycle with black border) and other prokaryotic taxa. Nine and six thaumarchaeotal OTUs were involved in the free-living and particle-attached community networks, respectively.

Supplementary Table S1 Sampling location and information for each sample

|  | Site | Latitude (ºN) | Longitude (ºE) | Size fraction | Sampling depth |
| --- | --- | --- | --- | --- | --- |
| Yellow  Sea | H1 | 36.9955 | 122.7449 | N | Surface |
|  | H2 | 37.0012 | 123.3797 | Y | SML, surface |
|  | H3 | 37.0026 | 123.9667 | Y | SML, surface |
|  | H8 | 38.4601 | 121.4242 | Y | SML, surface |
|  | H9 | 38.7490 | 122.0523 | Y | SML, surface |
|  | H10 | 38.7450 | 123.0239 | N | Surface |
|  | H11 | 38.7520 | 123.7983 | Y | SML, surface |
|  | B1 | 35.0084 | 120.5041 | N | Surface |
|  | B3 | 35.0001 | 122.2006 | N | Surface |
|  | B5 | 35.0010 | 123.9653 | N | Surface |
| East China Sea | D3 | 31.9012 | 122.9982 | Y | SML, surface |
|  | D5 | 32.4483 | 123.9648 | Y | SML, surface |
|  | F1 | 31.3310 | 122.9663 | Y | SML, surface |
|  | F3 | 31.3331 | 124.3542 | Y | SML, surface |
|  | F5 | 31.3335 | 126.1994 | Y | SML, surface |
|  | P1 | 31.3328 | 122.6059 | Y | Surface, 10 m, 30 m |
|  | P2 | 30.9178 | 123.1349 | Y | SML, surface, 10 m, 30 m, 50 m |
|  | P3 | 30.4008 | 123.6012 | Y | SML, surface, 10 m, 30 m, 60 m |
|  | P4 | 30.0002 | 124.3018 | Y | SML, surface, 10 m, 30 m, 50 m |
|  | P5 | 29.5984 | 124.9007 | Y | Surface, 10 m, 30 m, 50 m, 80 m |
|  | P6 | 29.1241 | 125.5023 | Y | Surface, 10 m, 30 m, 50 m, 75 m, 90 m |
|  | W1 | 28.5971 | 122.0044 | Y | SML, surface |
|  | W3 | 27.9027 | 122.8482 | Y | SML, surface |
|  | W4 | 27.5012 | 123.3510 | Y | SML, surface |
|  | T1 | 26.8662 | 120.4773 | Y | SML, surface |
|  | T3 | 26.2909 | 121.2002 | N | Surface |

N: size fragmented samples were not collected; Y: size fragmented samples were available; surface water indicates a water depth of 3 m.

Supplementary Table S2 Information of *Thaumarchaeota* OTUs and close neighbors

| OTU | Closely related sequences | 16S rRNA gene similarities | Lineage |
| --- | --- | --- | --- |
| OTU1488 | MZ048950.1 | 0.996 | Soil Crenarchaeotic Group |
| OTU2073 | KX648323.1 | 0.988 | *Nitrosopumilus*-like |
| OTU2211 | MG430416.1 | 0.989 | Soil Crenarchaeotic Group |
| OTU2233 | MF006045.1 | 0.993 | Soil Crenarchaeotic Group |
| OTU2236 | KF818592.1 | 0.982 | Soil Crenarchaeotic Group |
| OTU2245 | MG430477.1 | 0.993 | Soil Crenarchaeotic Group |
| OTU2300 | JX458357.1 | 0.989 | Soil Crenarchaeotic Group |
| OTU2408 | KX648354.1 | 0.989 | Water column B |
| OTU2418 | KX648349.1 | 0.989 | Water column B |
| OTU2744 | LN533573.1 | 0.993 | *Nitrosarchaeum*-like |
| OTU2989 | KX648325.1 | 0.989 | *Nitrosopelagicus*-like |
| OTU3141 | AF121988.1 | 0.996 | Water column B |
| OTU3244 | MN577002.1 | 0.996 | Soil Crenarchaeotic Group |
| OTU3585 | KX427649.1 | 0.985 | *Nitrosopelagicus*-like |
| OTU3909 | CP035425.1 | 0.989 | *Nitrosopumilus*-like |
| OTU4664 | MN577001.1 | 0.996 | Soil Crenarchaeotic Group |
| OTU4710 | KX061146.1 | 0.996 | Soil Crenarchaeotic Group |
| OTU4890 | MN308178.1 | 0.989 | undefined |
| OTU4920 | KX427804.1 | 0.989 | *Nitrosopelagicus*-like |
| OTU5351 | KT454272.1 | 0.989 | Water column B |
| OTU5377 | KX427766.1 | 0.985 | *Nitrosopelagicus*-like |
| OTU5384 | KX427887.1 | 0.989 | *Nitrosopelagicus*-like |
| OTU5396 | AB703777.1 | 0.982 | *Nitrosopelagicus*-like |
| OTU5402 | KT454278.1 | 0.989 | Water column B |
| OTU5406 | HQ529859.1 | 0.989 | Water column B |
| OTU5411 | HQ675852.1 | 0.985 | Water column B |
| OTU5419 | KX427766.1 | 0.989 | *Nitrosopelagicus*-like |
| OTU5420 | KX427657.1 | 0.989 | Water column B |
| OTU5422 | KX427579.1 | 0.989 | *Nitrosopelagicus*-like |
| OTU5441 | KX427658.1 | 0.989 | *Nitrosopelagicus*-like |
| OTU5460 | KT424646.1 | 0.989 | *Nitrosopelagicus*-like |
| OTU5486 | KT424509.1 | 0.985 | undefined |
| OTU6186 | CP035425.1 | 0.989 | *Nitrosopumilus*-like |

Supplementary Table S3 Environmental parameters of the seawater samples

| Sample | Depthm | Temperature/°C | Salinity | pH | NH_4_^+^  μmol/L | PO_4_^3-^  μmol/L | NO_3_^-^  μmol/L | NO_2_^-^  μmol/L | SiO_3_^2-^  μmol/L |
| --- | --- | --- | --- | --- | --- | --- | --- | --- | --- |
| H1 | 3 | 15.82 | 31.83 | 8.10 | N.D. | 0.05 | N.D. | 0.02 | 0.03 |
| H2 | 3 | 20.26 | 31.48 | 8.13 | 0.09 | 0.01 | 0.08 | 0.01 | N.D. |
| H3 | 3 | 21.84 | 31.75 | 8.12 | 0.06 | 0.03 | N.D. | 0.06 | N.D. |
| H8 | 3 | 19.44 | 31.79 | 8.06 | 0.11 | 0.04 | N.D. | 0.01 | N.D. |
| H9 | 3 | 20.68 | 32.13 | 8.13 | N.D. | 0.02 | N.D. | 0.03 | N.D. |
| H10 | 3 | 15.50 | 32.00 | 8.14 | 0.08 | 0.05 | N.D. | 0.01 | N.D. |
| H11 | 3 | 20.24 | 32.06 | 8.14 | N.D. | 0.02 | N.D. | 0.04 | N.D. |
| B1 | 3 | 21.91 | 30.88 | 8.14 | 0.30 | 0.03 | 0.89 | 0.04 | 0.09 |
| B3 | 3 | 21.53 | 32.40 | 8.14 | N.D. | 0.05 | N.D. | 0.00 | N.D. |
| B5 | 3 | 22.35 | 32.19 | 8.13 | 0.13 | 0.02 | 0.10 | 0.01 | N.D. |
| D3 | 3 | 24.23 | 25.18 | 8.32 | 0.92 | 0.15 | 18.14 | 0.86 | 22.78 |
| D5 | 3 | 27.18 | 30.54 | 8.30 | 0.19 | 0.07 | 2.09 | 0.28 | 3.15 |
| F1 | 3 | 24.79 | 29.36 | 8.10 | 0.51 | 0.09 | 9.97 | 0.84 | 4.03 |
| F3 | 3 | 26.26 | 31.68 | 8.27 | 0.27 | 0.08 | 0.62 | 0.05 | N.D. |
| F5 | 3 | 24.23 | 32.00 | 8.19 | N.D. | 0.02 | N.D. | N.D. | 6.68 |
| P1 | 3 | 23.28 | 26.51 | 8.12 | 0.40 | 0.18 | 22.94 | 0.81 | 26.26 |
| P2 | 3 | 27.02 | 31.74 | 8.29 | 0.18 | 0.05 | 0.25 | 0.03 | 5.49 |
| P3 | 3 | 27.76 | 31.74 | 8.29 | 0.08 | 0.05 | N.D. | 0.04 | 3.22 |
| P4 | 3 | 28.10 | 33.08 | 8.31 | 0.07 | 0.04 | N.D. | 0.05 | 1.14 |
| P5 | 3 | 26.92 | 33.85 | 8.20 | N.D. | 0.02 | N.D. | N.D. | 0.81 |
| P6 | 3 | 27.45 | 33.58 | 8.18 | 0.01 | 0.02 | N.D. | N.D. | 0.95 |
| W1 | 3 | 27.35 | 30.38 | 8.16 | 1.46 | 0.51 | 8.99 | 1.06 | 12.35 |
| W3 | 3 | 27.39 | 34.11 | 8.17 | 0.00 | 0.04 | N.D. | N.D. | 1.64 |
| W4 | 3 | 28.07 | 33.81 | 8.18 | 0.01 | 0.02 | 0.02 | N.D. | 0.27 |
| T1 | 3 | 26.28 | 34.02 | 8.15 | 0.05 | 0.21 | 0.48 | 0.40 | 3.42 |
| T3 | 3 | 27.05 | 33.97 | 8.18 | N.D. | 0.02 | N.D. | N.D. | 0.27 |
| P1_10m | 10 | 20.80 | 30.97 | 7.97 | 0.08 | 0.44 | 17.27 | 0.83 | 21.96 |
| P1_30m | 30 | 20.24 | 34.16 | 7.98 | N.D. | 0.68 | 11.37 | 0.14 | 18.87 |
| P2_10m | 10 | 25.76 | 32.61 | 8.25 | 0.22 | 0.05 | 0.80 | 0.04 | 5.28 |
| P2_30m | 30 | 19.27 | 34.31 | 8.00 | 0.02 | 0.78 | 12.27 | 0.30 | 19.68 |
| P2_50m | 50 | 19.25 | 34.32 | 8.00 | 0.03 | 0.75 | 12.31 | 0.28 | 19.88 |
| P3_10m | 10 | 27.51 | 32.13 | 8.29 | 0.19 | 0.05 | 0.08 | 0.03 | 3.13 |
| P3_30m | 30 | 23.80 | 34.33 | 8.10 | 0.05 | 0.06 | 1.49 | 0.16 | 4.97 |
| P3_60m | 60 | 20.50 | 34.45 | 8.04 | 0.01 | 0.52 | 8.57 | 0.15 | 14.78 |
| P4_10m | 10 | 28.10 | 33.08 | 8.31 | 0.04 | 0.04 | N.D. | 0.01 | 0.98 |
| P4_30m | 30 | 26.70 | 33.57 | 8.17 | 0.02 | 0.04 | N.D. | 0.02 | 0.97 |
| P4_50m | 50 | 23.57 | 34.31 | 8.11 | 0.01 | 0.23 | 1.99 | 0.10 | 6.88 |
| P5_10m | 10 | 26.90 | 33.84 | 8.20 | 0.04 | 0.01 | N.D. | N.D. | 0.77 |
| P5_30m | 30 | 23.72 | 34.01 | 8.21 | N.D. | 0.02 | N.D. | N.D. | 3.52 |
| P5_50m | 50 | 20.24 | 34.22 | 8.08 | N.D. | 0.48 | 7.37 | 0.06 | 11.96 |
| P5_80m | 80 | 19.86 | 34.26 | 8.08 | N.D. | 0.56 | 8.61 | 0.00 | 13.64 |
| P6_10m | 10 | 27.47 | 33.59 | 8.19 | 0.01 | 0.00 | N.D. | N.D. | 0.90 |
| P6_30m | 30 | 25.39 | 33.99 | 8.19 | N.D. | 0.02 | N.D. | N.D. | 0.93 |
| P6_50m | 50 | 20.78 | 34.16 | 8.21 | N.D. | 0.03 | 0.01 | N.D. | 11.96 |
| P6_75m | 75 | 18.58 | 34.51 | 8.07 | N.D. | 0.75 | 11.19 | 0.00 | 17.13 |
| P6_90m | 90 | 18.45 | 34.54 | 8.06 | N.D. | 0.77 | 11.27 | 0.02 | 18.18 |

N.D., not detected. Dissolved oxygen values are unpublished data and are used only for correlation analysis.

Other supplementary data

The sequence used for standard curve generation: TAGGCCCAATAATCATCCTGACCACTTGAGGTGCTGGTTTTACCGCGGCGGCTGACACCAGAACTTGCCCACCCCTTATTCATTAGTGGTTCTATGACTAACAAAAGGTTCCTTTAGCAGAAACCACTCGGATTAACCCTGTCGTGCTTTCGCACATTGCAAAGTTTTCTCGCCTGCTGCGCCCCATAGGGCCTGGGTCCGTGTCTCAGTACCCATCT
